# Gender-Specific Association of Cigarette Smoking with Spirometry and Cardiopulmonary Exercise Parameters in Elite Athletes—Impact of Cigarette Smoking in Elite Athletes

**DOI:** 10.3390/jcm15072677

**Published:** 2026-04-01

**Authors:** Giuseppe Di Gioia, Ilaria Menichini, Armando Ferrera, Alessandro Spinelli, Giacomo Canelli, Pier Giorgio Tiberi, Andrea Serdoz, Maria Rosaria Squeo

**Affiliations:** 1 Institute of Sport Medicine and Science, National Italian Olympic Committee, Largo Piero Gabrielli, 1, 00197 Rome, Italy; ilariamenichini@gmail.com (I.M.); armando.ferrera95@gmail.com (A.F.); alessandro.spinelli1@gmail.com (A.S.); piergiorgiotiberi@yahoo.com (P.G.T.); andreaserdoz@gmail.com (A.S.); mariarosaria.squeo@coni.it (M.R.S.); 2 Department of Clinical and Molecular Medicine, Sapienza University of Rome, 00189 Rome, Italy; 3 BIOMETRA Department, University of Milan, Via Festa del Perdono 7, 20122 Milan, Italy; giacomo.canelli@gmail.com

**Keywords:** olympic athletes, cardiovascular prevention, cigarette smoke, cardiopulmonary exercise test, sports medicine

## Abstract

**Background**: The impact of cigarette smoking on cardiopulmonary function in elite athletes remains poorly characterised. This study aimed to evaluate the prevalence of smoking and its effects on pulmonary and exercise performance parameters among top-level competitors across different sport disciplines. **Methods**: 1005 Olympic-level athletes participating underwent comprehensive pre-participation screening, including spirometry and cardiopulmonary exercise testing (CPET). Smoking status was determined according to WHO definitions. **Results**: Among the 1005 athletes (53.4% males; mean age 26 ± 8.8 years), 117 (11.6%) were current smokers, predominantly males (70.9%). No smokers were identified among endurance athletes. Compared to non-smokers (*n* = 679), smokers were older (28 ± 5.8 vs. 25.5 ± 10.4 years, *p* = 0.026) and more frequently involved in mixed and skill disciplines (*p* = 0.043 and *p* = 0.006, respectively). In male smokers, spirometry revealed lower FVC (97.2 ± 10.3% vs. 101.1 ± 11.7%, *p* = 0.006), FEV_1_ (97.3 ± 12.4% vs. 101.4 ± 10.3%, *p* = 0.002), and MVV (*p* = 0.010). CPET showed reduced functional capacity (W/kg, *p* < 0.0001), lower VO_2_max (38.5 ± 7.8 vs. 42.2 ± 6.8 mL/min/kg, *p* < 0.0001), decreased O_2_ pulse (*p* = 0.007) and lower first and second ventilatory thresholds (respectively *p* = 0.025; and *p* = 0.004), Similar but less pronounced reductions in VO_2_max were found in female smokers (*p* = 0.003). **Conclusions**: Chronic smoking is associated with early spirometric and ventilatory impairments in elite athletes associated with lower aerobic capacity, despite their high levels of fitness. These effects are more marked in males, while females may exhibit partial protection. These findings highlight the importance of integrating smoking cessation and respiratory monitoring into athlete health programs.

## 1. Introduction

Tobacco use remains one of the leading preventable causes of morbidity and mortality worldwide, responsible for over 8 million deaths annually according to the World Health Organisation [1]. According to European Society of Cardiology (ESC) guidelines on cardiovascular prevention, cigarette smoking is well-established as a major risk factor for cardiovascular and pulmonary disease, cancer, and premature death [2]. While the deleterious health consequences of smoking in the general population are well-documented, its impact on highly trained individuals, particularly elite athletes, is a subject of growing interest and concern.

Historically, it has been assumed that elite athletes, by virtue of their fitness, healthy lifestyles, and performance demands, would be protected against or unlikely to engage in cigarette smoking. However, recent surveys suggest that smoking prevalence, though lower than the general population, remains present in some athletic communities raging from approximately 15% to 25%, with considerable variability across sports, genders, and regions [3,4]. Despite growing awareness and anti-smoking campaigns targeting athletes, cultural and social factors—along with perceived stress-relief or weight-control benefits—may contribute to continued tobacco use even at high levels of competition [5,6].

The harmful effects of smoking on respiratory health are well-known. Tobacco smoke, through the generation of reactive oxygen species and pro-inflammatory mediators, induces airway inflammation, accelerates the decline of lung function, alters pulmonary diffusion, and promotes oxidative stress, even in asymptomatic individuals [7,8,9]. Importantly, spirometry may remain within normal ranges in smokers despite early airway remodelling and physiological impairment, especially in young or physically active populations. Notably, highly trained athletes generally exhibit lower baseline inflammatory markers and enhanced antioxidant defences compared to the general population [7]. However, smoking may attenuate these protective adaptations, potentially explaining the subclinical functional impairments observed in our cohort [7].

Despite the well-established detrimental effects of smoking in the general population, its impact on cardiopulmonary function in elite athletes remains poorly characterised. This population represents a unique physiological model, characterised by optimal cardiovascular and pulmonary performance, in which even subtle impairments may become detectable under high physiological demand.

Given these premises, the present study aims to explore the respiratory and ventilatory profiles of Olympic-level athletes with a history of cigarette smoking, using both spirometry and cardiopulmonary exercise testing (CPET), to better understand the long-term functional implications of tobacco use in this uniquely fit population.

## 2. Materials and Methods

### 2.1. Setting

The Institute of Sport Medicine and Science in Rome is the medical division overseen by the Italian National Olympic Committee, whose primary responsibility is medical evaluations of athletes selected to participate in the Olympic Games, World Championships, and Mediterranean Games. The research methodology employed in this investigation underwent scrutiny and approval by the Review Board of the Institute of Medicine and Sports Science and by the ethics committee (“Comitato Etico Territoriale Lazio Area 1”, approval number 0851/2024). All athletes involved in this study were comprehensively apprised of the nature and scope of the evaluation, and provided their informed consent in compliance with Italian Law and Institute protocols. All clinical data compiled from the study cohort are catalogued within an institutional database. The activities detailed herein were conducted in accordance with The Code of Ethics of the World Medical Association (Declaration of Helsinki).

### 2.2. Study Population

For the scope of this investigation, we enrolled 1005 elite athletes who participated in the Kraków 2023 European Games and Paris 2024 Olympic Games. Specifically, athletes were engaged in a broad spectrum of sport disciplines, classified into four groups, according to ESC guidelines [10]:

‒ skill (technical disciplines): archery, equestrian, golf, shooting, sailing, diving, equestrian sports.
‒ power (strength disciplines): weightlifting, Greco-Roman wrestling, judo, javelin, shot-putting, swimming (<800 mt), athletics (sprinting, shot putting and discus).
‒ mixed disciplines (alternate dynamic and strength components): soccer, volleyball, basketball, tennis, fencing, water polo, rhythmic gymnastics, taekwondo, badminton, beach volleyball, softball.
‒ endurance (primarily dynamic components): cycling, rowing, canoeing, triathlon, long-distance running, long-distance swimming (>800 mt), pentathlon.

Information regarding smoking habits, including duration of smoking and average number of cigarettes per day, was collected. Pack-years were not systematically available and therefore were not included in the analysis.

Inclusion criteria were:

(1) Olympic-level athletes evaluated at our Institute as part of the standardised pre-participation screening program for major international competitions; (2) availability of complete clinical, anthropometric, spirometric, and cardiopulmonary exercise testing (CPET) data; and (3) achievement of maximal or near-maximal exercise effort during CPET, defined by a respiratory exchange ratio (RER) > 1.05.

Exclusion criteria were:

(1) incomplete or missing smoking history data; (2) incomplete spirometry or CPET data; (3) failure to meet maximal effort criteria during CPET, and (4) presence of known cardiovascular, pulmonary, or systemic diseases potentially affecting exercise performance or pulmonary function.

Although no a priori sample size calculation was performed because of the observational design of the study, the final sample size (117 smokers and 679 non-smokers) was adequate to detect small-to-moderate effect sizes (standardised effect size ≈ 0.28) with 80% power at a two-sided alpha level of 0.05.

## 3. Methods

The athletes underwent a comprehensive, multidisciplinary pre-participation screening, which included a complete physical examination, resting electrocardiography (ECG), spirometry and CPET.

Blood pressure was measured in the sitting position before exercise testing, as recommended [2,11].

Body height and weight were obtained in each subject, and body mass index (BMI) was calculated as weight (kg)/height (m)^2^. Body surface area (BSA) was derived by the Mosteller formula [12].

CV risk factors were defined as follows:

- Family history for cardiovascular disease: fatal or non-fatal CV events and/or established diagnosis of CV disease in first-degree male relatives before 55 years, or female relatives before 65 years [11].

Participants’ smoking status was assessed through a standardised questionnaire administered at the time of enrollment. Subjects were classified according to the standard definitions proposed by the World Health Organisation (WHO) and the Centres for Disease Control and Prevention (CDCP), which distinguish current and never smokers based on lifetime cigarette consumption and current smoking behaviour [1].

Current smokers were defined as individuals who report having smoked at least 100 cigarettes in their lifetime and who currently smoke cigarettes (daily or occasionally). On the other side, smokers were never defined as individuals who report having smoked fewer than 100 cigarettes in their lifetime and who do not currently smoke. Moreover, information regarding the duration of smoking and the average number of cigarettes per day was collected.

A standard 12-lead ECG was performed in the supine position, and interpretation was made according to the international criteria for ECG interpretation in athletes [13].

Pulmonary function tests, including spirometry and maximal voluntary ventilation (MVV), were obtained in each athlete. These measurements were obtained by using a pulmonary function testing system (Quark PFT, Cosmed, Pavona, Italy). The procedures followed the standards recommended by the American Thoracic Society (ATS) and the European Respiratory Society (ERS) [14]. All measured variables were presented as absolute and percentage of the predicted values (adjusted for age, sex, BMI, and ethnicity) [15].

### 3.1. Cardio-Pulmonary Exercise Test

We performed CPET using a cycle ergometer (COSMED). The protocol, as previously described [16], began with a one-minute rest period, followed by a two-minute warm-up with no resistance. Afterwards, the workload increased progressively in increments of 15, 20, 25, or 30 watts using a ramp protocol, tailored according to the athlete’s gender and sports discipline, continuing until exhaustion. All athletes included in the analysis met the RER criterion of >1.05 before their maximal VO_2_ measurements were recorded [17]. Continuous ECG monitoring and recording (Quark T12x, COSMED) took place during the warm-up, exercise, and subsequent recovery period, which lasted for five minutes. Additionally, we utilised a breath-by-breath metabolimeter (Quark CPET; COSMED) to measure oxygen consumption and carbon dioxide production throughout the entire cardiopulmonary assessment. The ventilatory threshold (VT) was identified using both the V-slope and ventilatory equivalents methods. The respiratory compensation point (RCP) was determined by analysing the relationship between V’E and V’CO_2_ over time, following established standard criteria [17]. We recorded the following parameters at peak: VO_2_ in absolute (mL/min) and relative (mL/min/kg) values; Power (expressed in Watts); Heart rate (HR), beats per minute; Respiratory Quotient (RQ); Oxygen Pulse (VO_2_/HR). When reaching both the first ventilatory threshold (V1) and secondary ventilatory threshold (V2), measurements were conducted for: Power (expressed in Watts), VO_2_ (mL/min), and the slope of work efficiency (VO_2_/watts). All spirometry and CPET assessments were conducted using standardised protocols in accordance with international guidelines (ATS/ERS for spirometry and established CPET recommendations), with calibrated equipment and experienced operators [18].

### 3.2. Statistical Analysis

Categorical variables were represented as frequencies and percentages and compared using Fisher’s exact test or the Chi-square test, as appropriate. The normality of continuous variables was assessed, and these were reported as mean and standard deviation (SD). Non-parametric multiple comparisons between groups were carried out by Dunn’s test and the pairwise comparison method. A *p*-value of less than 0.05 was considered statistically significant. All statistical analyses were performed using SPSS version 29 (SPSS Inc., Chicago, IL, USA).

## 4. Results

We enrolled 1005 elite athletes, 537 (53.4%) males, mean age 26 ± 8.8 years old, practising different sporting disciplines, divided into skill (*n* = 163, 16.2%), power (*n* = 319, 31.7%), mixed (*n* = 314, 31.2%) and endurance (*n* = 209, 20.8%). Mean body weight was 73 ± 14.4 kg, mostly Caucasians (*n* = 960, 95.5%), while 325 (32.3%) had familiarity for CVD. In the overall cohort, 117 athletes (83 males, 70.9%) were defined as active smokers (11.6%). Twenty-five were past smokers. Smoking athletes consumed a mean of 7.3 ± 5.1 cigarettes\day, since a mean of 6.9 ± 6 years. Twenty-six of them smoked e-cigarettes, while 14 smoked more than 15 cigarettes a day. Similar habit history was found between male and female athletes, with males having 7 ± 6.4 years of smoking and females 6.7 ± 4.5 years, *p* = 0.772; also, the amount of cigarettes per day was similar (7.6 ± 5.5 cigarettes in males vs. 6.6 ± 4.1 cigarettes in females, *p* = 0.395). No smokers were present in the group of athletes practising endurance disciplines; athletes competed prevalently in mixed disciplines (56, 47.9%), then in skill (*n* = 35, 30%) and lastly in power sports (*n* = 26, 22.2%). In order to avoid confusing factors secondary to cardiac and pulmonary adaptations secondary to high-intensity training and considering the absence of smokers in the endurance group, this sport category was excluded from the non-smoking athletes used as the control group, obtaining a final number of non-smoking athletes of 679. As shown in Table 1, smokers were older (28 ± 5.8 years old vs. 25.5 ± 10.4 years old, *p* = 0.026) and had a higher prevalence of males (*n* = 83, 70.9% compared to 49.8% of non-smokers, *p* < 0.0001). In the smokers group, a higher prevalence of athletes practising mixed (*p* = 0.043) and skill (*p* = 0.006) disciplines was registered.

**Figure 1:**
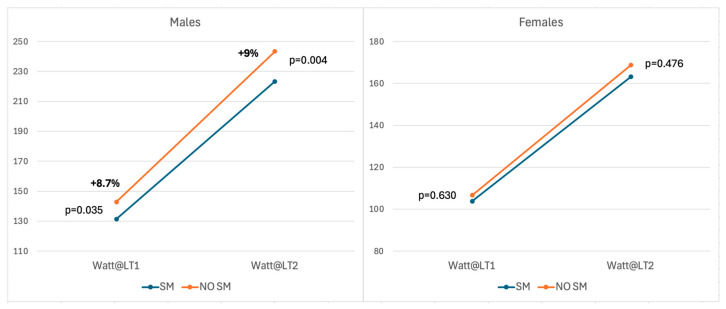
Watt reached at first (LT1) and second (LT2) ventilatory threshold in smokers (SM) and non-smokers (NO SM) athletes. (**Left**) panel: males. (**Right**) panel: females.

In Table 2, spirometry parameters were compared between smokers and the control group, also according to sex. In fact, different modifications were found between males and females. Males smokers presented lower values of FVC (in both L and %, respectively 5.68 ± 1.1 L and 97.2 ± 10.3%, compared to 5.91 ± 0.9 L and 101.1 ± 11.7%, *p* = 0.048 and *p* = 0.006), FEV1 (in L: 4.63 ± 0.8 L vs. 4.92 ± 0.7 L, *p* = 0.001 and in %: 97.3 ± 12.4% vs. 101.4 ± 10.3%, *p* = 0.002) and MVV (162 ± 31.4 L vs. 171 ± 27.5 L in non-smokers, *p* = 0.010). Differently, no significant variations in spirometry functional parameters were observed in females between the smokers and the control group. At CPET (Table 3), male smokers presented a lower functional capacity (expressed in W/kg, *p* < 0.0001), with a higher (but statistically not significant) prevalence of VEB (*p* = 0.089) associated with more VEB couplets (*p* = 0.004). Moreover, a lower VO_2_ max was also registered (38.5 ± 7.8 L/min/kg vs. 42.2 ± 6.8 L/min/kg in non-smokers, *p* < 0.0001) with lower VCO_2_ (*p* = 0.0004), lower O_2_ pulse (*p* = 0.007) and lower VE max (*p* = 0.048). Also, at first and secondary ventilatory threshold, smokers presented lower watts and VO_2_ (at first threshold, respectively, *p* = 0.035 and *p* = 0.025; at second threshold, respectively, *p* = 0.005 and *p* = 0.004), Figure 1. In the female group, a reduction in aerobic capacity was registered (*p* = 0.005) and a lower VO_2_ max (35.6 ± 5.4 mL/min/kg vs. 38.9 ± 6.3 mL/min/kg, *p* = 0.003).

## 5. Discussion

The present study provides novel insights into the impact of chronic cigarette smoking on pulmonary and ventilatory function in elite athletes. Despite the superior baseline fitness of this population, our data demonstrate that regular smokers—after a mean exposure of approximately seven years—exhibited measurable decrements in both resting spirometric parameters and exercise-related ventilatory performance. These findings support and extend recent evidence showing that smoking negatively affects cardiorespiratory physiology, even in young physically active individuals [19].

In particular, we observed significant reductions in FVC, FEV_1_, and MVV among male athletes who smoked, consistent with early obstructive changes in airflow mechanics. These findings echo prior literature describing the deleterious effects of smoking on lung function, including an accelerated decline in FEV_1_ and impaired maximal ventilatory capacity, even in young populations [7,19,20]. These alterations are primarily driven by smoking-induced airway inflammation, increased oxidative stress, and early structural remodelling of the small airways. Tobacco smoke promotes the release of pro-inflammatory mediators and reactive oxygen species, leading to epithelial damage, mucus hypersecretion, and reduced airway calibre. In parallel, oxidative stress may impair respiratory muscle function and reduce ventilatory efficiency during exercise. Together, these mechanisms may contribute to a progressive decline in expiratory flow and maximal ventilatory capacity, even before overt airflow limitation becomes clinically evident [8,20].

Moreover, the reduction in ventilatory parameters during CPET, including VO_2_ max and ventilatory thresholds (VT1/LT1 and VT2/LT2), suggests aerobic performance in smokers, aligning with the observations made by Borrelli et al. (2025) [19]. These findings are further supported by studies indicating that smokers, even in the absence of airflow obstruction, show reduced skeletal muscle endurance and aerobic capacity during exercise. Exercise is known to exert important immune-regulating effects, including the reduction in systemic inflammation and enhancement of antioxidant capacity. In the context of cigarette smoke exposure, regular physical activity may partially mitigate smoke-induced inflammatory responses by modulating cytokine production and oxidative stress pathways. However, these protective adaptations may not fully counterbalance the harmful effects of tobacco smoke, particularly at the level of the airways and skeletal muscle. This may explain why, despite their high level of training, smoking athletes in our cohort still exhibited reduced ventilatory and aerobic performance [21].

The mechanisms behind these impairments are multifactorial. Cigarette smoke contains a complex mixture of oxidants and inflammatory agents that contribute to airway inflammation, increased mucus production, and early structural remodelling of small airways [8]. This has been observed not only in clinical pathology but also in imaging and functional studies of smokers with preserved spirometry [7]. Additionally, oxidative stress and muscle dysfunction have been documented in healthy smokers, which may explain the reduced O_2_ pulse and VO_2_ kinetics during exercise [8,20,22].

A particularly interesting and novel aspect of our study is the observed sex difference in response to smoking. While male athletes showed significant reductions in both spirometric and ventilatory parameters, female smokers exhibited relatively preserved function. No significant differences were found in spirometric values between smoking and non-smoking women, and although VO_2_ max was lower in female smokers, the magnitude of difference was smaller than in males. These findings suggest that female athletes may benefit from biological or hormonal protective factors. Studies on gender and lung development suggest that sex hormones may influence inflammatory and oxidative pathways, possibly modulating lung vulnerability to smoke exposure [23]. Estrogens, in particular, exert well-established anti-inflammatory and antioxidant effects, modulating endothelial function, oxidative stress, and immune responses. Within the emerging framework of cardiovascular–endocrine–metabolic interactions, hormonal regulation plays a central role in shaping systemic resilience to external stressors, including cigarette smoke exposure. This integrated response may partially explain the attenuated pulmonary impairment observed in female athletes [24].

However, the absence of significant spirometric impairment in female smokers should not be interpreted as evidence of immunity to tobacco-related harm. Functional decline was still evident at CPET, and longer-term exposure may eventually lead to similar patterns of deterioration. Moreover, evidence suggests that ventilatory impairment may precede airflow obstruction in otherwise healthy young smokers [7,19].

This study also adds to the body of evidence suggesting that smoking prevalence among elite athletes, although lower than in the general population, is not negligible. In our cohort, smokers were primarily found in skill and mixed sports. These trends mirror previous findings on the prevalence of tobacco use in professional athletes [4] and should inform targeted prevention efforts.

Our findings carry relevant clinical implications. Sports medicine practitioners should be aware that even modest smoking habits can result in subclinical pulmonary and ventilatory impairment in athletes. Pre-participation evaluations should incorporate not only standard spirometry but also exercise testing where possible, particularly in those with known or suspected smoking history. Smoking cessation interventions should be integrated into athlete health programs regardless of performance level, with additional emphasis on male athletes who appear more vulnerable to early functional decline.

In summary, our findings indicate that chronic smoking is associated with early pulmonary function decline and reduced ventilatory performance in elite athletes, despite their otherwise optimal fitness. These effects were more pronounced in males, while female athletes demonstrated relative preservation of spirometric parameters, potentially due to sex-related biological factors. This study underscores the need for smoking prevention and cessation strategies within athletic populations, and for the routine inclusion of respiratory health surveillance in elite sports medicine.

### Limitations

Several limitations should be considered when interpreting the findings of this study.

First, the vast majority of the athletes included in this analysis were of Caucasian ethnicity. While this reflects the demographic composition of the national teams involved, it limits the generalizability of the findings to more diverse athletic populations. Ethnic differences in lung size, airway reactivity, and susceptibility to smoking-related pulmonary damage have been documented in the literature [25], Future studies should aim to include a more ethnically heterogeneous cohort to improve external validity.

Second, this was a single-centre study conducted within the context of a specialised sports medicine facility. While the testing protocols were standardised and carried out by experienced personnel using high-quality equipment, the monocentric nature of the study may introduce centre-specific biases related to selection, testing environment, or procedural nuances. Multicenter studies involving various geographic regions and institutions would provide a more robust and representative overview of smoking-related functional changes in athletes.

Third, the athletes included in the study were assessed at different time points across their competitive season. Consequently, variations in training load, environmental exposure, recovery status, and physiological conditioning at the time of testing may have influenced CPET and spirometry results. Although all athletes were part of Olympic-level programs and maintained a consistently high weekly training volume (approximately 25 h/week), subtle differences in periodisation may have introduced variability. Nevertheless, the elite status of the cohort implies a minimal likelihood of detraining phases, supporting the reliability of the observed performance metrics. Moreover, specific training modalities, including respiratory muscle training, could have influenced the outcomes and should be considered in future studies [26].

Fourth, the CPET was performed on a cycle ergometer in all cases to ensure methodological consistency and standardisation of measurements. While this allowed for uniform data collection and error distribution, it may have disadvantaged athletes from non-cycling disciplines, particularly those less familiar with cycling biomechanics. However, given that all participants were evaluated using the same modality, any performance limitation introduced by the testing modality would be evenly distributed across groups, preserving the internal validity of the comparisons.

Fifth, the cross-sectional design of the study inherently limits causal inference. Although we observed consistent and biologically plausible associations between cigarette smoking and reductions in both spirometric and ventilatory parameters, the temporal relationship between exposure and physiological decline cannot be definitively established.

Beyond the observed associations, our findings raise the hypothesis that cigarette smoking may interfere with the physiological adaptations to exercise, potentially blunting the beneficial effects of training at both pulmonary and peripheral levels. In this context, smoking could lead to a form of subclinical functional impairment, not detectable at rest but emerging under exercise conditions. This concept may have important implications for athlete evaluation, suggesting that CPET could serve as a sensitive tool to identify early functional alterations in otherwise asymptomatic individuals.

However, it remains uncertain whether the observed impairments are directly attributable to tobacco use or influenced by other unmeasured confounders, such as environmental exposure, respiratory infections, or genetic predispositions. Prospective longitudinal studies, ideally including smoking cessation follow-up, would be necessary to assess causality and potential reversibility of functional changes.

Sixth, smoking exposure was assessed through self-reported history, which is subject to recall and social desirability bias, especially in elite athletes, where smoking may be stigmatised or underreported. While structured interviews were used to enhance reporting accuracy, future studies should consider the use of objective biomarkers, such as urinary cotinine levels or exhaled carbon monoxide, to quantify both acute and chronic tobacco exposure more reliably. Biomarker-based assessment would also allow for dose–response analyses and better stratification of risk based on smoking intensity and duration.

Furthermore, in the present study, we did not perform a formal analysis of measurement error indices such as coefficient of variation (CV), smallest worthwhile change (SWC), or standard error of measurement (SEM), as the study was based on a retrospective analysis of clinically collected data.

However, all spirometry and CPET assessments were conducted using standardised protocols in accordance with international guidelines (ATS/ERS for spirometry and established CPET recommendations), with calibrated equipment and experienced operators. This approach ensures a high level of reproducibility and reliability of the measurements.

In light of these limitations, the findings of this study should be interpreted with caution. Nonetheless, the consistency of our results with existing literature and the homogeneity of testing protocols across a highly trained athletic cohort lend credibility to the observed associations and support further investigation into the subclinical impact of smoking in elite sports settings.

## 6. Conclusions

The results of this study suggest that chronic cigarette smoking is associated with significant impairments in pulmonary function and ventilatory performance, even in Olympic-level athletes with otherwise optimal aerobic capacity. These alterations include reductions in FEV_1_, FVC, MVV, VO_2_ max, and ventilatory thresholds, in line with previous findings in young and physically active populations.

Our findings also contribute to the discussion of sex differences in smoking-related effects. Male smokers exhibited more pronounced reductions in both spirometric and CPET-derived parameters, while female smokers showed relatively preserved baseline lung function despite a mild reduction in VO_2_ max. These results may reflect anatomical or hormonal differences in pulmonary responses to smoke exposure, as previously suggested by studies on sex-based modulation of pulmonary inflammation and remodelling.

From a public health and sports medicine perspective, it is concerning that cigarette smoking remains present among elite athletes. Previous research has identified non-negligible smoking rates among athletes involved in team or skill-based sports, and our findings confirm that tobacco use continues to pose a tangible risk within this subgroup.

In conclusion, this study reinforces the evidence that cigarette smoking causes early detrimental effects on respiratory function and exercise tolerance in elite athletes. These changes are clinically relevant, impact performance, and justify the implementation of preventive strategies and systematic respiratory screening in professional sports contexts.

Sports medicine professionals, coaches, and athletic trainers should be aware that even moderate smoking may negatively impact performance and long-term health.

## Figures and Tables

**Table 1:** Main clinical, anthropometric and sporting disciplines differences between smokers and non-smokers athletes.

	Non Smokers	Smokers	*p*
N (%)	679	117	
**Age, years**	**25.5 ± 10.4**	**28 ± 5.8**	**0.026**
**Male, *n* (%)**	**338 (49.8)**	**83 (70.9)**	**<0.0001**
**Weight, kg**	**72.9 ± 14.4**	**78 ± 15.3**	**0.0005**
**BMI, kg/m^2^**	**23.2 ± 2.9**	**24 ± 3.1**	**0.020**
SBP, mmHg	114.4 ± 10.8	116.9 ± 9.1	0.137
DBP, mmHg	70.4 ± 6.9	71.5 ± 6.3	0.123
Rest HR, bpm	58.4 ± 10.4	58.7 ± 10.1	0.750
**Skill, *n* (%)**	**128 (18.8)**	**35 (30)**	**0.006**
**Power, *n* (%)**	**293 (43.1)**	**26 (22.2)**	**<0.0001**
**Mixed, *n* (%)**	**258 (38)**	**56 (47.9)**	**0.043**
Endurance, *n* (%)	-	0 (0)	

Abbreviations: BMI: body mass index; DBP: diastolic blood pressure; HR: heart rate; SBP: systolic blood pressure.

**Table 2:** Comparison of main spirometry parameters in smokers and non-smokers, also divided by sex.

	Males, *n* = 421			Females, *n* = 375		
	Non Smokers	Smokers	*p*	Non Smokers	Smokers	*p*
N (%)	338 (80.3)	83 (19.7)	-	341 (90.9)	34 (9.1)	-
VC, L	5.78 ± 0.9	5.63 ± 1	0.213	4.21 ± 0.7	4.35 ± 0.7	0.309
VC, %	98.6 ± 13.1	96.5 ± 11.5	0.177	100.8 ± 15.1	99 ± 13.1	0.496
IC, L	4.1 ± 0.8	4.1 ± 0.7	0.623	3.05 ± 0.6	3.15 ± 0.7	0.373
IC, %	106.9 ± 18.6	104.2 ± 16.9	0.245	115.1 ± 20.4	108.7 ± 20.2	0.087
**FVC, L**	**5.91 ± 0.9**	**5.68 ± 1.1**	**0.048**	4.31 ± 0.7	4.31 ± 0.7	0.953
**FVC, %**	**101.1 ± 11.7**	**97.2 ± 10.3**	**0.006**	102.8 ± 13.4	98.1 ± 10.5	0.062
**FEV1, L**	**4.92 ± 0.7**	**4.63 ± 0.8**	**0.001**	3.68 ± 0.5	3.7 ± 0.5	0.845
**FEV1, %**	**101.4 ± 10.3**	**97.3 ± 12.4**	**0.002**	102.6 ± 12.7	100 ± 10.4	0.234
TI	83.6 ± 6.8	82.3 ± 7.7	0.120	85.7 ± 6.7	86.4 ± 6.7	0.595
**MVV, L**	**171 ± 27.5**	**162 ± 31.4**	**0.010**	121.9 ± 20.9	124.5 ± 24.5	0.508

Abbreviations: FEV1: forced expiratory volume in 1 s; FVC: forced vital capacity; IC: inspiratory capacity; MVV: maximal voluntary ventilation; TI: Tiffeneau index; VC: vital capacity.

**Table 3:** Comparison of main cardiopulmonary test parameters in smokers and non-smokers, also divided by sex.

	Males, *n* = 421			Females, *n* = 375		
	Non Smokers	Smokers	*p*	Non Smokers	Smokers	*p*
N (%)	338 (80.3)	83 (19.7)	-	341 (90.9)	34 (9.1)	-
W/kg	**3.39 ± 0.6**	**3.1 ± 0.6**	**<0.0001**	**3.1 ± 0.5**	**2.8 ± 0.5**	**0.005**
SBP peak, mmHg	182.4 ± 19	181.1 ± 17	0.599	164.2 ± 15.5	162.2 ± 12.2	0.478
DBP peak, mmHg	82 ± 7.6	82.9 ± 7.4	0.323	77.8 ± 7.2	79 ± 5.8	0.374
HR max, bpm	167.5 ± 11.5	165.5 ± 12.6	0.181	**170.9 ± 11.2**	**165.6 ± 9.2**	**0.019**
VEB, *n* (%)	60 (17.7)	22 (26.5)	0.089	58 (17)	4 (11.8)	0.440
Polymorphic, *n* (%)	12 (3.5)	2 (2.4)	0.759	6 (1.7)	0 (0)	0.668
Couplets, *n* (%)	**2 (0.6)**	**4 (4.8)**	**0.004**	5 (1.5)	0 (0)	0.475
SVEB, *n* (%)	27 (8)	10 (12)	0.263	21 (6.2)	4 (11.8)	0.223
VO_2_ max, mL/min/kg	**42.2 ± 6.8**	**38.5 ± 7.8**	**<0.0001**	**38.9 ± 6.3**	**35.6 ± 5.4**	**0.003**
VCO_2_	**3802.1 ± 678.8**	**3491.8 ± 782.1**	**0.0004**	2685.8 ± 485	2553.1 ± 695.2	0.147
RQ	**1.13 ± 0.07**	**1.15 ± 0.07**	**0.006**	1.10 ± 0.07	1.11 ± 0.08	0.381
O_2_ pulse, mL/beat	**20.7 ± 3.9**	**19.4 ± 4.4**	**0.007**	14.6 ± 2.7	14.6 ± 2.9	0.987
VE max, L/min	**116.3 ± 25.6**	**110.3 ± 21.1**	**0.048**	84.7 ± 16.9	81.6 ± 13.6	0.301
BR, L/min	32.3 ± 15	32.6 ± 16.8	0.891	29.7 ± 18.1	35.9 ± 13.6	0.056
TV, L	2.97 ± 0.5	2.9 ± 0.5	0.294	2.12 ± 0.4	2.11 ± 0.4	0.816
Watt @LT1	**142.9 ± 43.6**	**131.4 ± 46.3**	**0.035**	106.7 ± 33.6	103.8 ± 29.9	0.630
VO_2_ @LT1	**1971.1 ± 535.9**	**1819.5 ± 536.1**	**0.022**	1552.6 ± 399.3	1464.7 ± 345.3	0.217
VE/VCO_2_@LT1	25.3 ± 2.8	25.7 ± 2.3	0.254	27.4 ± 2.9	27.3 ± 2.8	0.861
Watt @LT2	**243.3 ± 55.3**	**223.3 ± 55.1**	**0.005**	168.7 ± 40.9	163.1 ± 41.4	0.476
VO_2_ @LT2	**3000.3 ± 670.9**	**2753.6 ± 611**	**0.004**	2141.9 ± 497.6	2097.8 ± 503.4	0.644
VE/VCO_2_@LT2	26.7 ± 3.1	27 ± 3.1	0.390	28.2 ± 3.2	29.4 ± 3.6	0.065
VO_2_/Watt	12.5 ± 1.5	12.8 ± 1.7	0.159	12.8 ± 1.5	12.8 ± 1.4	0.801
VE/VCO_2_ slope	25.7 ± 3.2	25.7 ± 3.6	0.288	26.9 ± 4	27 ± 4.2	0.933

Abbreviations: BR: breath rate; DBP: diastolic blood pressure; HR: heart rate; LT1: first ventilatory threshold; LT2: second ventilatory threshold; RQ: respiratory quotient; SBP: systolic blood pressure; SVEB: supraventricular extra-beats; VE: ventilation; VE/VCO_2_: ventilatory efficiency; VEB: ventricular extra-beats; W: watt; VT: tidal volume.

## Data Availability

De-identified participant data are available upon reasonable request from the corresponding author.
